# The Impact of a Spanish Online and Telephone Intervention for Caregivers of Veterans Post-Stroke: Findings on Caregiver Burden

**DOI:** 10.3390/healthcare13243202

**Published:** 2025-12-08

**Authors:** Janet Lopez, Sara Snyder, Tatiana Orozco, Heather Foulke, Melanie Orejuela, Alexa Farriss, Naiomi Rivera-Rivera, Nathaniel Eliazar-Macke, Keryl Motta-Valencia, I. Magaly Freytes

**Affiliations:** 1 College of Nursing, University of Central Florida, Orlando, FL 32827, USA; 2 VA North Florida/South Georgia Veterans Health System, Gainesville, FL 32608, USAivette.freytes@va.gov (I.M.F.); 3 College of Health and Human Performance, University of Florida, Gainesville, FL 32608, USA; 4 VA Caribbean Healthcare System, San Juan, PR 00921, USA; 5 VA Geriatric Research Education Clinical Center, Gainesville, FL 32608, USA

**Keywords:** stroke, informal caregivers, Hispanic/Latino, problem-solving intervention

## Abstract

**Background/Objectives:** Hispanic caregivers report higher levels of burden and poorer mental health compared to non-Hispanic caregivers. While problem-solving interventions have shown promise in improving stroke caregiver and patient well-being, they have not been specifically tailored or tested with Hispanic stroke caregivers. This study aimed to evaluate the effectiveness of a telephone- and online-based problem-solving intervention that was culturally adapted for Spanish-speaking Hispanics on caregiver burden. **Methods:** A two-arm parallel randomized clinical trial (NCT03142841) with repeated measures was conducted with 210 Hispanic caregivers of veterans who had experienced a stroke within the past year. Participants were recruited from three VA medical centers. The intervention consisted of 8-telephone sessions using online materials conducted by a trained interventionist. Participants in the standard group received usual VA resources they would have received as part of routine care such as stroke-related information. Chi-square tests and regression analyses were used to assess outcomes at 9 and 21 weeks. **Results:** Most participants were female (88%), college-educated (49%), and spouses or partners of a veteran (46%). Caregivers in the intervention group showed significantly greater reductions in caregiver burden (*p* = 0.011; *p* < 0.001) compared to the standard care group. **Conclusions:** Similarly to others reporting positive findings with non-Hispanic caregivers, these findings suggest that the problem-solving intervention can potentially alleviate Hispanic Spanish-speaking stroke caregivers’ burden by improving coping skills, problem-solving, and social support. Further research is needed to address gaps in effective interventions and promote equitable care for this population.

## 1. Introduction

Stroke remains a leading cause of long-term disability, often resulting in significant physical, cognitive, and emotional impairments [1]. In the aftermath of a stroke, informal caregivers, frequently family members, assume critical roles in supporting survivors of stroke’s recovery and managing their needs [2,3]. Family caregivers are instrumental in the recovery of stroke-survivors helping reduce inpatient stays in nursing homes and hospitals [4]. Thus, informal caregiving has been identified as a major component of public health [3].

Stroke caregivers report feeling underprepared for their role [5]. Caregiving is associated with elevated risks for psychological distress among caregivers, particularly depression and burden [3,4,6]. Caregiver burden can also have a negative impact on the quality of care provided to care recipients [7]. These challenges are further compounded when caregivers belong to linguistically and culturally diverse populations, such as Spanish-speaking communities, where access to culturally sensitive support services is often limited [8,9,10].

There is ample evidence about the negative impact of caregiver burden [7,11,12]. Additionally, caregiver burden is recognized as a major contributor to the institutionalization of stroke survivors and is associated with reduced functional recovery following a stroke [13,14]. As caregivers’ health declines, so does their ability to provide effective care. Moreover, Hispanic caregivers often face elevated burden and psychological distress compared to non-Hispanic caregivers [3,8,15,16].

Minority groups have been shown to practice higher levels of caregiving than their white counterparts, receive less professional support, and experience poorer health outcomes [5,17,18]. However, there is little research on how stroke caregivers from underserved communities cope and adjust to this role. Caregiver education and support have shown to be beneficial in helping facilitate the transition home for individuals following a stroke [19,20,21]. Studies have shown that providing caregivers with information, support, and skills-training has the potential to reduce negative caregiver outcomes and increase the likelihood that stroke survivors remain at home [2,19].

Existing interventions for caregivers of older adults have shown promise in reducing psychological distress and improving coping strategies [2,5,22]. However, minority populations, including Hispanic caregivers, are underrepresented in stroke caregiver interventions studies [23]. No culturally tailored interventions have been developed specifically for Spanish-speaking Hispanic stroke caregivers, especially those caring for veterans. One study recommended developing culturally appropriate mental health interventions for stroke caregivers from diverse racial and ethnic backgrounds [8]. Moreover, most existing studies on Hispanic stroke caregivers are observational or descriptive in nature [8,9,10,15,24,25], rather than intervention-based, highlighting a lack of evidence on effective, culturally responsive strategies. Evidence show Hispanic caregivers report greater physical burden and overall needs [15]. Hispanic stroke caregivers, in particular, have more information needs than non-Hispanic White caregivers [9]. Sociocultural differences also play a role in how recovery is experienced for both survivors and caregivers, highlighting the need for policies and system-level changes to support underserved communities in post-stroke care [10].

In summary, existing stroke caregiver interventions often underrepresent Hispanic populations and are not culturally tailored, which can reduce accessibility and exacerbate disparities for Spanish-speaking caregivers. To address this gap, we evaluated the impact of a culturally adapted Spanish problem-solving intervention that combines telephone support and web-based education on caregiver burden. We hypothesize that stroke caregivers receiving the intervention would experience less burden at 9 and 21 weeks post-test compared to those in the standard care group. To our knowledge, this is the first study to test a problem-solving intervention specifically developed for Spanish-speaking caregivers of veterans post-stroke. This study addresses key gaps in caregiver intervention research, testing a culturally and linguistically adapted approach that considers the cultural context and thus, provides a more pertinent solution for Spanish-speaking participants. The results of this study are promising and support the positive impact of tailored interventions on Spanish-speaking Hispanic caregivers of veterans post-stroke.

## 2. Materials and Methods

We analyzed data collected from the Recursos Educativos Sirviendo a Cuidadores en Español (RESCUE en Español) study, a two-arm parallel randomized clinical trial with three assessment points (i.e., baseline and 9 week and 21 weeks post-test) [26]. The RESCUE en Español intervention consists of eight telephone sessions with a trained interventionist and is based on the relational/problem-solving model of stress developed by D-Zurilla and Nezu [27]. The intervention can be summarized by the acronym COPE (Creativity, Optimism, Planning, and Expert Information) [28]. During the sessions caregivers are encouraged to have an optimistic attitude and to realize that effective problem solving can be learned and successfully used. Effective problem solving involves several steps: (1) identifying and defining problems; (2) prioritizing problems; (3) selecting the highest priority problem; (4) gathering expert information; (5) setting realistic goals; (6) listing all possible solutions; (7) choosing the best solution; and (8) evaluating the plan. Thus, the intervention empowered caregivers to identify and solve problems while receiving education [28].

### 2.1. Sample and Procedures

Informal caregivers of veterans who survived a stroke were recruited from three Veterans Health Administration (VHA) medical centers (i.e., Tampa and Orlando, Florida and Puerto Rico). Potential participants were identified through healthcare provider referrals and list of patients with a stroke diagnosis (ICD-9: 430–438; ICD-10: I60.0–I69.998). Prior to the initiation of the project, procedures were established, and staff were trained to address cases of severe caregiver stress or crisis.

Caregivers were eligible to participate in the study if they self-identified as the primary caregiver of a veteran diagnosed with stroke within the past year as confirmed in the electronic health record. Additional inclusion criteria included self-identifying as Hispanic, Spanish language preference, internet and phone access, moderate-to-high stress (Spanish Perceived Stress Scale ≥ 1) [29], and willingness to be randomized. The care recipient (Veteran) was required to have at least two ADL deficits (Stroke Impact Scale-16) ≤ 74 [30] or a new/worsening neurological condition. Caregivers who were actively enrolled in other studies or caring for veterans with a life expectancy of less than five months were excluded. Caregivers of veterans residing in assisted living facilities were also excluded.

Caregivers who met inclusion criteria and completed consent procedures were enrolled in the study. We obtained a waiver of written consent because the intervention is conducted by phone to reduce participant burden. Trained research staff conduct consent procedures by phone using a written statement mailed in advance to explain study details and obtain oral permission for recording. Upon enrollment, caregivers were mailed a caregiver workbook that included the instructions to access various materials on the RESCUE en Español website, contact information for study staff, problem-solving slides, a problem-solving diary, and paper for easy note taking. Ten to 14 days after mailing the workbook, participants were contacted to schedule baseline data collection.

Caregivers were randomly assigned to one of two study arms using a randomization procedure, to maintain group assignment balance over time [31]. The randomization scheme was randomly generated, and treatment allocations were given to the unblinded members of the research team in sealed opaque envelopes. When a caregiver consented to participate in the study, the project coordinator opened a new envelope that revealed the caregiver’s group assignment. All data collectors and study principal investigators were blinded to group assignments, and access to assignment-related information was restricted through a secure, password-protected database for unblinded team members. Only the statistician and study coordinator were unblinded to group assignment.

Data collectors were experienced and trained on study-specific assessment tools, which were conducted over the telephone. Caregivers’ responses were entered into an online database and checked for accuracy by another member of the team post-test assessments were conducted at 9 and 21 weeks post-baseline.

Intervention adherence was ensured using a standardized manual. The principal investigator and project coordinator reviewed notes to evaluate adherence, recording any deviations on a standardized form. To ensure treatment fidelity, the interventionists received comprehensive training and a detailed intervention manual. Intervention fidelity monitoring through observation was conducted on 10% of the intervention sessions using a detailed standardized checklist. To support caregivers’ adherence to the intervention, we emphasize the importance of keeping up with study procedures during the first session made and reminder calls prior to the sessions.

This study was approved by the University of Florida Institutional Review Board and the VA Research and Development Committee at all three sites, pre-registered on ClinicalTrials.gov (NCT03142841), and conducted in accordance with the Declaration of Helsinki. For more detailed information on study procedures, including sample size calculation and randomization procedures, please refer to the published protocol [26].

### 2.2. Measures/Instruments

A demographic questionnaire developed and used in previous studies [32] was translated into Spanish by the research team and used to gather information on caregiver characteristics (e.g., gender, age, employment, etc.).

Caregiver burden was measured by the Spanish 22-item Zarit Burden Interview (ZBI-22) [33], which assesses subjective and objective burden experienced by caregivers over the last month. Items fall into five categories (health, well-being, finances, social life, relationship with impaired person) with response options indicating frequency of burden 0 = Never, 1 = Rarely, 2 = Sometimes, 3 = Quite frequently, 4 = Nearly always), and scores ranging from 0 to 88, with a score 61 or higher considered a high burden. The Spanish 22-item ZBI has shown good internal consistency in multiple previous studies typically reporting a Cronbach’s alpha around 0.88–0.92 [33,34,35,36].

### 2.3. Data Analysis

To describe the participant sample, we ran summary-descriptive statistics for demographics. We tested the primary hypotheses—whether the amount of change in burden over time was influenced by the intervention—using generalized least squares (GLs) regression models for repeated measures. The model tested for the main effect of group (intervention vs. standard care), main effects of time (baseline vs. follow-up times (9 and 21 weeks), and interactions between group and time (group × baseline-vs-post 1, group × baseline-vs-post 2). While the assumptions of constant error variance and independence of errors were relaxed with this modeling, we examined the data for outliers and tested for normality of regression residuals using boxplots and Q-Q plots; no remarkable assumptions violations were noted.

## 3. Results

**Figure 1:**
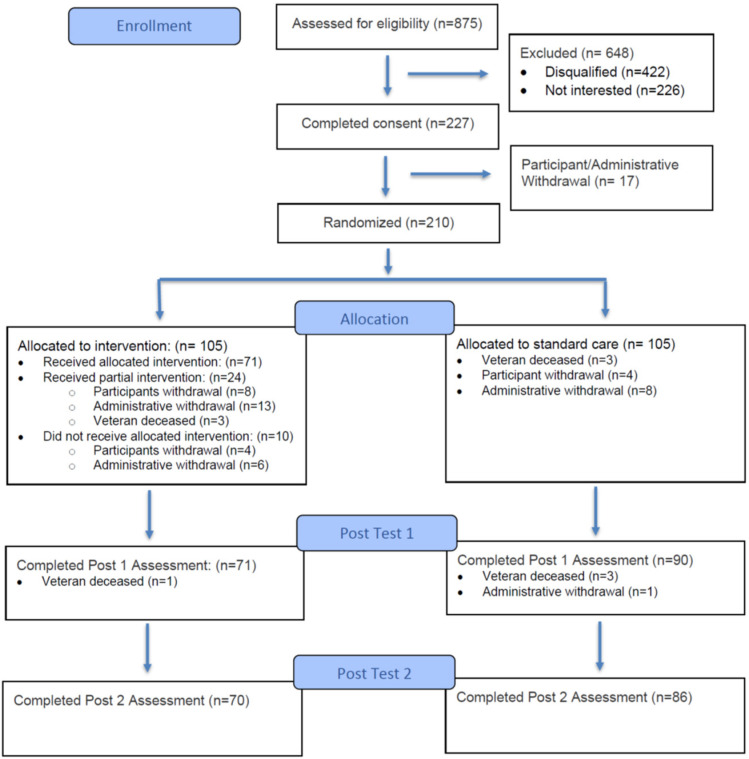
Flow of participants.

A total of 875 caregivers were assessed for eligibility; 648 were excluded, and 17 withdrew prior to randomization. Two hundred ten participants were enrolled and randomly assigned to either the intervention group (n = 105) or standard care group (n = 105). One hundred fifty-six caregivers completed both post assessments at 9 and 21 weeks: 70 in the intervention group and 86 in the standard care group. While not all participants completed the study, we have retained the available data for participants who were lost to follow-up and analyzed them according to an intent-to-treat analysis principle. The participant flow is shown in Figure 1.

We also conducted comparisons of baseline demographics between caregivers who completed all data collection assessments (i.e., “completers,” regardless of which intervention group they were randomized to) and caregivers who were lost to follow-up (i.e., “withdrawals”) using non-parametric tests. Specifically, continuous variables were compared using Wilcoxon rank sum tests, while categorical variables were compared using chi-square and Fisher’s exact tests.

Overall, the majority of participants identified as Hispanic (99%) and female (88%), with an average age of 59. Nearly half (46%) reported being the spouse or partner of the Veteran. Comparison of baseline demographics between completers vs. withdrawals showed that caregivers who withdrew from the study were significantly more likely to have been randomized to the intervention group, with 65% of withdrawals having been assigned to the intervention group and the remaining 35% having been assigned to the standard card group. Caregivers in the completers group had a higher proportion of participants who had a college degree or higher. Additionally, baseline demographic comparisons revealed that caregivers in the intervention group were significantly more likely to have a college degree or higher. See Table 1 for characteristic of caregivers.

### Caregiver Burden

Results from the burden hypothesis testing revealed significant interaction effects indicating that changes in burden over time varied by group. Analysis revealed two significant Group × Time interactions. The interaction between group and Post 1 (9 weeks) vs. Baseline contrast was statistically significant (*p* = 0.011), as was the interaction between group and the Post 2 (21 weeks) vs. Baseline contrast (*p* < 0.001). These findings indicate that changes over time differed significantly between groups at both post-intervention timepoints relative to baseline. Examination of group means showed that the intervention group experienced a modest 4.8-point reduction in burden from baseline to one week post intervention and an additional 1.2-point decrease by 21 weeks. In contrast, the standard care group’s burden scores remained consistent across all three timepoints, with no change exceeding 1 point. These findings highlight the intervention’s effectiveness in reducing caregiver burden throughout the post-intervention period. See Table 2 for caregiver burden summary statistics and Table 3 for burden regression results.

To supplement the unstandardized results, we computed standardized regression coefficients by scaling and centering Zarit burden scores and refitting an analogous GLS model with the standardized outcome. These coefficients express the group × time effects in standard-deviation units and can facilitate comparison with conventional effect-size benchmarks. However, effect-size reporting in generalized least squares and longitudinal mixed-effects models is an area without strong consensus, particularly given correlated within-subject errors and time-specific variances. Accordingly, we report both unstandardized coefficients—which reflect clinically meaningful point differences on the Zarit scale—and standardized coefficients to provide complementary perspectives on the magnitude of the intervention effect.

Although we did not anticipate effects related to caregiver education, there was a baseline difference in education level between groups, as noted previously. We therefore added education to the primary regression model as a sensitivity check. Including education did not alter the magnitude or significance of the intervention effects (group × time estimates changed by <0.1 points). This difference in baseline education likely reflects chance imbalance.

Though the sample size calculation is detailed elsewhere [26] and based on the primary outcome of caregiver depression, we note that the target sample size was initially 290, which allowed for an anticipated 10% attrition rate. To evaluate whether the final analytic sample retained adequate statistical power, we computed the minimum detectable effect (MDE) for the primary group × time interaction using parameters estimated from the GLS model (residual variance and the unstructured within-subject covariance matrix). The MDE represents the smallest intervention-related difference in burden trajectories that the study could detect with 80% power at α = 0.05, given the actual sample size and correlation structure. Using the model-derived estimates, the MDE ranged from 3.5 to 3.7 points on the Zarit Burden Interview, corresponding to approximately 0.25–0.30 SD units. This approach is preferred over post hoc power calculations, which are not meaningful once statistical significance has been evaluated. Because the estimated MDE fell below the magnitude of the observed group × time effect, the study retained sufficient power to detect clinically meaningful changes despite attrition.

Overall, the results demonstrate that the intervention had a sustained positive impact on caregiver burden. Caregivers who received the intervention experienced significant improvements in burden compared to those who received standard care.

## 4. Discussion

This study examined the impact of the culturally adapted RESCUE en Español intervention on caregiver burden and among Hispanic caregivers of veteran stroke survivors. As hypothesized, the participants who received the intervention demonstrated reductions in caregiver burden at both 9 weeks and 21 weeks follow-ups compared to those receiving standard stroke education and support.

It is important to note that the Zarit Burden Interview (ZBI-22) assesses both subjective and objective caregiver burden, with scores of 61 or higher indicating high burden. In our study, the intervention group started with a mean score of 29.2 and the Standard Care group with 30.4, well below the high-burden threshold. Highlighting these baseline values helps contextualize the observed 4.8-point reduction in the intervention group, showing that even moderate initial burden can be meaningfully reduced. This is important because interventions that reduce burden at these levels may prevent escalation to high caregiver stress, potentially improving caregiver well-being and the quality of care provided.

While these findings demonstrate evidence that this intervention can meaningfully reduce caregiver burden, these data do not necessarily point to a specific causal mechanism. It may be that the intervention reduced burden through its impact on problem solving, coping skills, and ability to obtain social support. On the other hand, caregivers in the intervention group may have felt pressure to report lower burden after the intervention, in order to please data collector or because they enjoyed the calls with the interventionist. Regression to the mean is another possible explanation, though this seems unlikely, given that the standard care group showed consistent scores across three measured timepoints, while the intervention group showed reduction between the baseline and 9-week follow-up timepoints, and showed sustained reduction in burden at the 21-week timepoint. In other words, data show evidence for consistent between-group differences emerging over time, rather than showing an immediate drop at 9 weeks only.

Differential attrition is another possible explanation for these findings. For example, it is possible that the standard care group withdrawals would have shown reduced burden over time, had they remained in the study; such an attrition pattern may create the appearance of a spurious group difference in trajectories. This explanation also seems unlikely, however, as lower burden would likely make study compliance and continuation easier, while higher burden seems more likely to be associated with difficulty in study compliance and continuation.

Consistent with existing literature, our findings suggest that problem-solving interventions provide an effective option to support stroke caregivers, including those caring for individuals with complex comorbidities such as veterans. Our findings contribute to the current evidence regarding caregiver burden, which is inconclusive. While some studies report significant improvements, others show minimal or no change [37,38,39]. Similarly to our study, which demonstrated sustained improvements in caregiver outcomes 21 weeks post-intervention, Grant et al. (2002) [38] reported that the effects of problem-solving interventions were maintained over the long term [40]. However, other research has found that these outcomes tend to diminish within one year [41], suggesting variability in the durability of intervention effects. More research, including long-term outcomes, is needed to clarify the maintenance of intervention effects.

Problem-solving skill-based interventions have previously been found to be effective and have been integrated successfully into health interventions for Spanish-speaking Hispanic populations [42,43]. A culturally adapted problem-solving interventions designed for Spanish-speaking caregivers of adults with neurological conditions have demonstrated reductions in caregiver burden, improvements in depressive symptoms, and enhancements in caregiver–recipient relationships [44]. Nonetheless, no prior intervention has been specifically tailored for Spanish-speaking caregivers of veterans post-stroke. Compared to other interventions, RESCUE en Español incorporated a more comprehensive cultural adaptation- not merely translation- integrating culturally relevant values and beliefs to enhance engagement and effectiveness. Thereby, this study directly addressed a gap identified in a recent systematic review, which found few caregiver programs included culturally adapted content, tailored interventions modifications or targeted recruitment strategies [45]. Therefore, it is possible that the improvements may be, in part, attributed to the program’s cultural and linguistic adaptation for Spanish-speaking, Hispanic caregivers. By integrating teaching problem-solving strategies with stroke online educational materials and telephone support, the intervention better addressed culturally specific needs and preferences of these caregivers. This finding may help explain the improvements observed in caregiver burden.

Technology plays an important role in improving post-stroke caregiving [46,47]. It is essential to design interventions that put caregivers first to make them effective and practical. Using technology-based psychosocial interventions can help caregivers feel more confident and skilled [46,47]. It can also reduce anxiety and depression among family members caring for stroke survivors [46,47]. Different ways to deliver support were used, including a mix of in-person and telehealth visits, standalone telehealth, online learning, and telephone support [46,47]. Our study includes delivery via telephone, which is one of the preferred method for the Hispanic population regarding health communication [48,49]. We also made our materials accessible online through the RESCUE en Español website. Unlike other studies, our use of technology was able to demonstrate a reduction in burden [46].

Supporting the well-being of caregivers has broader implications beyond individual outcomes. By reducing caregiver burden, interventions like RESCUE en Español may enhance caregivers’ capacity to provide sustained care at home, potentially delaying or preventing institutionalization of veterans [7]. This not only improves quality of life for both caregivers and care recipients, but may reduce reliance on formal healthcare services, easing the burden on the healthcare system. Thoughtful integration of culturally adapted interventions into practice can further support caregivers. Investing culturally tailored caregiver support programs is therefore a strategic approach to strengthening home-based care and promoting health system sustainability, particularly within populations that experience disparities in access and outcomes. Future research should evaluate these approaches in diverse settings and examine long-term outcomes.

### Limitations

Several factors should be considered when interpreting the findings of this study. The focus on caregivers of veterans with the great majority of participants from Puerto Rican descend, limits applicability to the broader population of Hispanic stroke caregivers. Future research with larger, more diverse samples of Spanish-speaking Hispanic caregivers is warranted. While positive outcomes were observed at the 12-week follow-up, this relatively short timeframe limits the understanding of the intervention’s long-term effects. Understanding the maintenance of the effect for RESCUE en Español can only be confirmed with extended follow-up. Additionally, data collection was impacted by significant external events, including natural disasters and an island-wide blackout, which delayed the study timeline and may have influenced participant responses. These interruptions may have introduced variability in participant engagement. We acknowledge these events as potential sources of random variability and have noted this as a study limitation. Furthermore, it is unclear how these events impacted the study arms and data collection across sites, and this should be examined in future studies. Some caregivers did not complete data collection as scheduled due various reasons (e.g., missed calls, illness, natural disaster, etc.). Finally, reliance on self-reported measures may introduce bias, particularly in telephone-based assessments, where social desirability may influence responses, especially given the cultural norms around communication in this population.

Attrition between randomization and the first post-intervention assessment was higher in the intervention group compared to standard care. Most withdrawals in the intervention arm were due to being “too busy,” followed by perceived lack of benefit from participating. Administrative reasons, such as inability to contact, eligibility changes, and veteran death—also contributed substantially. Standard care withdrawals were fewer and primarily related to contact issues and time constraints.

Despite the intervention’s cultural and linguistic tailoring, these findings highlight persistent barriers: time burden, perceived relevance, and engagement challenges. For future implementation and scalability, strategies should include flexible, low-burden delivery formats, early demonstration of value, and robust outreach protocols. Planning for attrition and integrating culturally responsive approaches with practical scheduling solutions will be essential to sustain engagement on a larger scale.

## 5. Conclusions

The RESCUE en Español intervention was specifically adapted for Hispanic Spanish-speaking stroke caregivers of veterans, a historically underserved population within stroke caregiving research. The positive findings from our study underscore the value of culturally sensitive caregiver interventions, particularly problem-solving approaches, suggesting the potential benefit of addressing caregiver challenges and reducing caregiver burden. This study contributes to efforts aimed at identifying sustainable, scalable interventions that can be integrated into routine clinical practice to better support caregivers of stroke veterans and improve access to quality care for minority populations. Future research directions could include multi-site studies, objective measures, and examination of long-term effects evaluation. Achieving these outcomes requires a deeper understanding of the sociocultural factors influencing health behaviors in Hispanic communities. Due to interethnic differences among Hispanic caregivers, further research should replicate this work with larger, more diverse samples across geographic location and national origin. Additionally, future implementation should focus on reducing time burden, reinforcing perceived benefit, and strengthening engagement strategies to sustain participation and scalability. Continued research is critical to addressing existing knowledge gaps and ensuring future interventions are both effective and equitable.

## Figures and Tables

**Table 1:** Caregiver Characteristics—Overall Sample.

Characteristic	Overall N = 210 ^1^	Intervention N = 105 ^1^	Standard Care N = 105 ^1^	*p*-Value ^2^
**Age**	59 (12)	60 (12)	59 (11)	0.3
**Gender**				0.8
Male	25 (12%)	13 (12%)	12 (11%)	
Female	185 (88%)	92 (88%)	93 (89%)	
**Marital Status**				0.3
Single	32 (15%)	21 (20%)	11 (10%)	
Married	136 (65%)	62 (60%)	74 (70%)	
Living together with partner	14 (6.7%)	6 (5.8%)	8 (7.6%)	
Divorced or separated	25 (12%)	14 (13%)	11 (10%)	
Widowed	2 (1.0%)	1 (1.0%)	1 (1.0%)	
Refused	0 (0%)	0 (0%)	0 (0%)	
Unknown	1 (0.5%)	1 (0.01%)	0 (0%)	
**Ethnicity**				0.9
Hispanic	208 (99%)	104 (99%)	104 (99%)	
Not Hispanic	1 (0.5%)	0 (0%)	1 (1.0%)	
Unknown	1 (0.5%)	1 (1%)	0 (0%)	
**Race**				0.2
White	101 (52%)	51 (53%)	50 (52%)	
Black	18 (9.3%)	13 (13%)	5 (5.2%)	
Multi-racial	61 (32%)	27 (28%)	34 (35%)	
Other	11 (5.7%)	6 (6.2%)	5 (5.2%)	
Pacific Island Native	2 (1.0%)	0 (0%)	2 (2.1%)	
Unknown	17 (8.1%)	8 (7.61%)	9 (8.6%)	
**Education**				0.046
High school degree and lower	41 (20%)	24 (23%)	17 (16%)	
Some college/vocational	67 (32%)	39 (37%)	28 (27%)	
College degree and up (e.g., Postgraduate)	102 (49%)	42 (40%)	60 (57%)	
**Employment**				0.2
Full-time	42 (20%)	15 (15%)	27 (26%)	
Part-time	18 (8.7%)	10 (9.7%)	8 (7.7%)	
Retired	79 (38%)	43 (42%)	36 (35%)	
Unemployed	68 (33%)	35 (34%)	33 (32%)	
Unknown	3	2	1	
**Income**				0.2
Up to USD 20,000	81 (39%)	46 (45%)	35 (34%)	
USD 20,001–USD 50,000	91 (44%)	43 (42%)	48 (47%)	
USD 50,001 and higher	34 (17%)	14 (14%)	20 (19%)	
Unknown	4	2	2	
**Relation to Veteran**				0.4
Spouse/Partner	107 (51%)	50 (48%)	57 (54%)	
Child	70 (33%)	35 (33%)	35 (33%)	
Other	33 (16%)	20 (19%)	13 (12%)	

^1^ Mean (SD); n (%); ^2^ Wilcoxon rank sum test; Pearson’s Chi-squared test; Fisher’s exact test.

**Table 2:** Summary Statistics for Caregiver Burden.

Caregiver Burden	N	Mean	SD	SE	95% CI
Standard Care Baseline	102	30.4	7.6	0.8	[28.9, 31.9]
Standard Care Post 1 (9 weeks)	89	30.6	5.9	0.6	[29.4, 31.8]
Standard Care Post 2 (21 weeks)	84	30.8	6.7	0.7	[29.3, 32.3]
Intervention Baseline	104	29.2	6.9	0.7	[27.9, 30.5]
Intervention Post 1 (9 week)	70	24.4	6.6	0.8	[22.8, 26]
Intervention Post 2 (21-week)	70	23.2	7.0	0.8	[21.5, 24.9]

N = Sample size; SD = Standard deviation; SE = Standard error; CI = Confidence interval.

**Table 3:** Fixed effects from the generalized least squares model predicting Caregiver Burden.

Effect	β	B	SE B	95% CI	*p*-Value
Intercept	0.14	30.52	1.42	[27.72, 33.31]	<0.001 **
Time 1: Baseline vs. Post 1 (9-week)	0.00	0.07	1.10	[−2.09, 2.24]	0.947
Time 2: Baseline vs. Post 2 (21-week)	0.03	0.47	1.21	[−1.90, 2.95]	0.695
Group: Intervention vs. Standard Care	0.09	−1.33	2.01	[−5.28, 2.62]	0.508
Group × Time 1	0.28	−4.18	1.64	[−7.40, −0.97]	0.011 *
Group × Time 2	0.40	−5.87	1.79	[−9.38, −2.36]	0.001 **

β = Standardized Coefficient, B = Unstandardized Coefficient, SE B = Standard Error for Unstandardized Coefficient; CI = Confidence interval for Unstandardized Coefficient; * *p* < 0.05, ** *p* < 0.01.

## Data Availability

Final datasets from the proposed research will not be shared outside the VA, except as required under the Freedom of Information Act (FOIA). The data are not publicly available due to privacy or ethical restrictions. Only de-identified, aggregated data will be shared in publications and presentations. The data collected is specific to the study and has no applicable use outside this research context, therefore it will not be available upon request.

## Abbreviations

The following abbreviations are used in this manuscript:

ADL	Activities of Daily Living
COPE	Creativity, Optimism, Planning and Expert Information
EHR	Electronic Health Record
ICD	International Classification of Diseases
IRB	Institutional Review Board
PSS-10	Perceived Stress Scale-10
RESCUE en Español	Recursos Educativos Sirviendo a Cuidadores en Español
SIS-16	Stroke Impact Scale-16
U.S.	United States
VA	Veterans Affairs
VA HSRD	Veteran Affairs Healthcare Systems Research and Development
VHA	Veterans Health Administration
ZBI-12	12-item Short-form Zarit Burden Interview
